# Residents' Wayfinding Challenges and Environmental Interventions in a Care Home

**DOI:** 10.1093/geroni/igab046.2036

**Published:** 2021-12-17

**Authors:** Habib Chaudhury, Shelby Elkes

**Affiliations:** Simon Fraser University, Vancouver, British Columbia, Canada

## Abstract

This study evaluated the role of the built environment on residents’ wayfinding behaviours at Louis Brier Home in Vancouver, British Columbia, Canada. The goal of this study was to explore baseline mobility challenges for the residents traveling between their bedrooms and social spaces. In response to this, low-cost environmental interventions were proposed and implemented to support safe and independent wayfinding for the residents. The project consisted of three phases. First phase involved a mixed methods approach using behavior mapping and spatial observations of the residents interacting with their physical environment, combined with one focus group with the staff members. In the second phase, researchers presented actionable environmental interventions for the care home administration to consider and implement. The final phase involved post-implementation behaviour mapping, spatial observations and a focus group session. The implemented environmental interventions influenced in improved resident wayfinding and orientation in the long-term care home.

